# Sports Activity and Arrhythmic Risk in Cardiomyopathies and Channelopathies: A Critical Review of European Guidelines on Sports Cardiology in Patients with Cardiovascular Diseases

**DOI:** 10.3390/medicina57040308

**Published:** 2021-03-25

**Authors:** Giovanni Volpato, Umberto Falanga, Laura Cipolletta, Manuel Antonio Conti, Gino Grifoni, Giuseppe Ciliberti, Alessia Urbinati, Alessandro Barbarossa, Giulia Stronati, Marco Fogante, Marco Bergonti, Valentina Catto, Federico Guerra, Andrea Giovagnoni, Antonio Dello Russo, Michela Casella, Paolo Compagnucci

**Affiliations:** 1 Department of Biomedical Science and Public Health, Cardiology and Arrhythmology Clinic, University Hospital “Ospedali Riuniti Umberto I-Lancisi-Salesi”, Marche Polytechnic University, 60100 Ancona, Italy; u.falanga@pm.univpm.it (U.F.); cipollettalaura@gmail.com (L.C.); clapson@hotmail.it (M.A.C.); ginogrifoni@gmail.com (G.G.); giuseppe.ciliberti@ospedaliriuniti.marche.it (G.C.); alessiaurbinati@hotmail.it (A.U.); alessandro.barbarossa@ospedaliriuniti.marche.it (A.B.); giulia.emily.stronati@gmail.com (G.S.); guerra.fede@gmail.com (F.G.); antonio.dellorusso@gmail.com (A.D.R.); paolocompagnucci1@gmail.com (P.C.); 2 Department of Radiology, University Hospital “Ospedali Riuniti Umberto I-Lancisi-Salesi”, Marche Polytechnic University, 60100 Ancona, Italy; marco.fogante89@gmail.com (M.F.); a.giovagnoni@staff.univpm.it (A.G.); 3 Department of Clinical Sciences and Community Health, University of Milan, 20100 Milan, Italy; bergmar21@gmail.com; 4 Centro Cardiologico Monzino, Istituto di Ricovero e Cura a Carattere Scientifico, 20100 Milan, Italy; catto.valentina@gmail.com; 5 Department of Clinical, Special and Dental Sciences, Cardiology and Arrhythmology Clinic, University Hospital “Ospedali Riuniti Umberto I-Lancisi-Salesi”, Marche Polytechnic University, 60100 Ancona, Italy; michelacasella@hotmail.com

**Keywords:** sport, athletes, sudden cardiac death, cardiomyopathies, channelopathies

## Abstract

The prediction and prevention of sudden cardiac death is the philosopher’s stone of clinical cardiac electrophysiology. Sports can act as triggers of fatal arrhythmias and therefore it is essential to promptly frame the athlete at risk and to carefully evaluate the suitability for both competitive and recreational sports activity. A history of syncope or palpitations, the presence of premature ventricular complexes or more complex arrhythmias, a reduced left ventricular systolic function, or the presence of known or familiar heart disease should prompt a thorough evaluation with second level examinations. In this regard, cardiac magnetic resonance and electrophysiological study play important roles in the diagnostic work-up. The role of genetics is increasing both in cardiomyopathies and in channelopathies, and a careful evaluation must be focused on genotype positive/phenotype negative subjects. In addition to being a trigger for fatal arrhythmias in certain cardiomyopathies, sports also play a role in the progression of the disease itself, especially in the case arrhythmogenic right ventricular cardiomyopathy. In this paper, we review the latest European guidelines on sport cardiology in patients with cardiovascular diseases, focusing on arrhythmic risk stratification and the management of cardiomyopathies and channelopathies.

## 1. Introduction

Cardiovascular diseases are a significant cause of sudden cardiac death (SCD) in athletes, and exercise may be a trigger for fatal arrhythmias, with an annual incidence of about 1/200,000 [1]. The identification of athletes with cardiomyopathies or channelop-athies has important implications for participation in sports activities, especially with regard to the prevention of fatal events. Exercise intensity represents a critical variable in athletes with cardiovascular diseases, and it is related to metabolic equivalents (METs) or percentage of maximal aerobic capacity (VO2 max) during sports activity. Athletes can be divided into competitive athletes (exercising > 6 h/week) and recreational athletes (exercising > 4 h/week). In the following sections, we present a practical approach to athletes with heart diseases that have a potentially increasing risk of fatal arrhythmias during sports activity, according to the latest European Society of Cardiology (ESC) guidelines [2].

## 2. Discussion

### 2.1. Hypertrophic Cardiomyopathy

Hypertrophic cardiomyopathy (HCM) is defined as a cardiomyopathy characterized by a wall thickness ≥15 mm in one or more left ventricular (LV) myocardial segments that is not explained by other loading conditions. The diagnosis of HCM in athletes can be challenging due to LV adaptation to high workload, which is characterized by a variable degree of wall hypertrophy: 3 months detraining may be helpful to distinguish physiological hypertrophy (usually reversible) from pathological hypertrophy (generally persistent). HCM is the first cause of SCD in athletes and, in the past, a diagnosis of HCM implicated an absolute contraindication to sports practice. Nowadays, given the not so low prevalence of this condition, and the fact that the risk of SCD during sports activity is not as high as anticipated, risk stratification is necessary. Risk assessments for athletes with hypertrophic cardiomyopathy should include history, with particular emphasis on previous unexplained syncope and exercise-induced symptoms [3]. Presence of non-sustained ventricular tachycardia (NSVT) during 24/48-h ECG monitoring confers a higher risk of sudden cardiac death [4]. Echocardiographic evaluation should include left ventricular (LV) wall thickness, left ventricular outflow tract (LVOT) gradient, and left atrial diameter in parasternal long axis (PLAX) view. LVOT obstruction is defined as a peak pressure gradient >30 mmHg at rest or during stress. Cardiac magnetic resonance (CMR) seems to be useful for risk stratification of SCD; myocardial fibrosis is present in about 75% of patients with HCM, and a large area of myocardial scars seems to be related with poor outcomes [5]. However, at this time, there is no consensus to use this parameter as a guide for ICD implantation or sports contraindication [6,7]. Patients with exercise-induced symptoms, arrhythmias, or hypotension during an exercise treadmill test should avoid competitive sports. In addition, exercise stress echocardiography is useful for detecting LVOT obstruction in patients with exercise-related symptoms. Recently, some pharmacological agents (calcium channel blockers, angiotensin receptor blockers, ranolazine) were studied, trying to induced LV reverse remodeling, increase exercise performance, and improve quality of life in patients with HCM; none of these therapies proved useful, although ranolazine was associated with a reduction of premature ventricular complex (PVC) burden [8]. Most of these evaluations are included in HCM [9]. Based on this score, patients with a risk score >6% of SCD at 5 years should be implanted with an ICD. However, it should be recognized that this score is validated in non-athlete patients, and we currently lack a specific score for risk stratification of SCD in athletes. Athletes with HCM should be evaluated every 6 months, or earlie, in case new symptoms develop. In athletes with a positive genotype for HCM but without phenotypic characteristics, follow-up should be based on patients’ age and type of exercise.

### 2.2. Arrhythmogenic Cardiomyopathy

The influence of sports activity is particularly marked in patients with arrhythmogenic cardiomyopathy (ACM), in which intense physical exercise can drive disease progression [10]. Irrespective of the estimated risk of SCD, there is scientific evidence supporting the concept that high intensity exercise and competitive sports should be avoided. Actually, this recommendation is based on studies concerning cohorts of athletes with arrhythmogenic right ventricle cardiomyopathy (ARVC). We know nowadays that there exist a subtype of ACM that can affect mainly the left ventricle (ALVC), or left and right ventricles equally (ABVC) [11,12]. A history of unexplained syncope and exercise-induced symptoms are the most relevant risk markers of SCD [13,14]. twenty-four/forty-eight hour ECG monitoring is the cornerstone of SCD risk assessments. Th presence of NSVT or PVC burden >1000/24 h identify athletes at high risk of ventricular arrhythmias (VA). Right ventricle (RV) and LV function should be assessed by echocardiography or CMR; RV dilatation and function are relevant in risk stratification for arrhythmic risk. Patients with ACM engaged in low or moderate intensity sports should be evaluated every year, or every 6 months for adolescents and young adults.

### 2.3. Dilated Cardiomyopathy

A diagnosis of dilated cardiomyopathy is challenging due to LV adaptation in athletes: it is not uncommon to observe LV dilatation and mild dysfunction in endurance sports athletes [15]. The assessment of diastolic function (normal in the case of an athlete’s heart), stress echocardiography (increase contractility in the case of an athlete’s heart), and Vo2 max during cardiopulmonary exercise test can be helpful to distinguish these conditions. Hyperabeculation is also a characteristic of an athlete’s heart, and this condition can be challenging to distinguish from LV non-compaction cardiomyopathy; however, in the absence of LV dysfunction or high PVC burden, sports activity is not contraindicated. SCD in patients with dilated cardiomyopathy (DCM) is mostly related to LV systolic function and New York Hear Association (NYHA) class [16]. Genetic evaluation has a role; both lamin A/C and filamin C mutations are related to a higher risk of SCD. The evaluation of sports eligibility should include a history of unexplained syncope, palpitation, VAs during exercise treadmill test, and LV function and morphology by echocardiography. High intensity exercise and competitive sports should be avoided in patients with these characteristics. Annual follow up is recommended for most patients.

### 2.4. Myocarditis

Myocarditis is an important cause of SCD in athletes [2]. Myocarditis falls into the category of inflammatory cardiomyopathies and can cause LV dysfunction and fatal arrhythmias. Inflammatory cardiomyopathies include various forms such as cardiac sarcoidosis, autoimmune myocarditis in the context of connective tissue disease, giant cell myocarditis, or eosinophilic cardiomyopathies. Most myocarditis recognize a viral etiology. In addition, the abuse of performance-enhancing drugs such as cocaine, methamphetamine, or ephedrine can incite myocardial inflammation [17,18,19]. Clinical presentation is highly variable and includes supraventricular or ventricular arrhythmias, advanced atrioventricular block, heart failure, cardiogenic shock, or sudden cardiac death. In the acute phase, exercise could result in accelerated viral replication, increased inflammation, and cellular necrosis. Patients with acute myocarditis most commonly have polymorphic and irregular ventricular arrhythmias, based on more dynamic arrhythmogenic substrates characterized by active inflammation and necrosis of cardiomyocytes. Patients with prior myocarditis are characterized by monomorphic ventricular arrhythmias based on scar-related reentry, which may be difficult to control with drug therapy [20,21]. Echocardiographic evaluation and especially cardiac magnetic resonance (CMR) imaging are essential in the diagnostic work-up. The extent and distribution of late gadolinium enhancement (LGE) are independent predictors of cardiovascular events during follow-up, and patients with anteroseptal LGE localization have a worse outcome [22]. Endomyocardial biopsy (EMB) remains the gold standard for diagnosis and differentiates between the various types of inflammatory processes and guiding therapy; it is appropriate to use anti-inflammatory drugs and immunosuppressive therapies only in patients with virus-negative acute or chronic active myocarditis [23]. Electroanatomic voltage mapping (EVM) increases the sensitivity and specificity of EMB by disclosing areas characterized by abnormal voltage, and is currently regarded as the preferred guide to EMB [24,25]. EVM in myocarditis also has a prognostic role, and patients with areas of abnormal bipolar voltage spanning more than 10% of endocardial surface have been found to be at increased risk of major arrhythmias [26]. Abstention from sporting activity, including leisure activities, is recommended for at least 6 months as there is an increased risk of sudden cardiac death in the acute phase. Return to sports activity should only be considered in asymptomatic patients with normal troponin levels and markers of inflammation, normal left ventricular systolic function on both echocardiogram and cardiac magnetic resonance imaging, absence of active inflammation or fibrosis on CMR, and absence of complex ventricular arrhythmias on 24 h ECG monitoring and exercise stress testing. In asymptomatic patients with LGE persisting for more than 3–6 months, in the absence of ventricular systolic dysfunction and complex ventricular arrhythmias both during exercise and prolonged 24/48 h ECG monitoring, with normal biomarkers, return to sport activity should be assessed on a case-by-case basis. Patients with persistent systolic dysfunction and a large area of scarring (>20% LGE) should abstain from exercise programs and sports activities that require moderate or intense levels of activity. Focal inferoseptal RV insertion LGE is common in athletes and represents an adaptation to exercise and should not be confused with myocarditis [27]. Recently, athletes with Coronavirus disease 2019 (COVID-19) who were asymptomatic or had mild symptoms were evaluated with CMR, and the prevalence of myocarditis appeared modest (3%). A recent expert consensus recommended the return of asymptomatic or mildly symptomatic COVID-19 positive athletes to physical activity after 2 weeks of recovery in the absence of cardiac biomarker and imaging abnormalities. It is to be borne in mind that patients with COVID 19 are at high arrhythmic risk in the acute phase of the infection [28]. In hospitalized COVID-19 positive athletes, troponin evaluation and the use of second level tests such as CMR are recommended. In this setting, echocardiography plays an important role, both in the acute and the recovery phases for LV function assessment, and global longitudinal strain (GLS) appears to be a potential marker of subtle myocardial dysfunction [29]. If there is evidence of myocarditis, the return to sports should be assessed as detailed above. The return to sports activity must also consider the severity of the SARS-Cov-2 infection [30,31].

### 2.5. Wolff-Parkinson-White Syndrome

The risk of SCD in patients with Wolff-Parkinson-White syndrome (WPW) ranges from 0.15 to 0.2%, and usually occurs during exercise or emotional stress, when increased sympathetic activity can lead to atrial fibrillation with rapid ventricular activation over the accessory pathway, potentially precipitating ventricular fibrillation [32]. In order to non-invasively establish the risk of sudden cardiac death, exercise stress testing may be considered; the disappearance of pre-excitation at peak exercise indicates low risk of rapid conduction over the accessory pathway (AP). Ablation of the AP is recommended in both competitive and recreational athletes with documented arrhythmias. In athletes with asymptomatic pre-excitation or infrequent and well-tolerated arrhythmias, an electrophysiology study is recommended to assess the risk of sudden cardiac death. In the event of a high-risk findings (AP anterograde effective refractory period <250 ms or <220 ms during isoproterenol infusion, pre-exited R-R interval during AF <250 ms, multiple APs), ablation of the AP is indicated [33]. If the athlete refuses ablation or the procedure is high-risk (e.g., anteroseptal AP), the decision to participate in sports activity must be discussed on a case-by-case basis, including the use of drug therapy (e.g., class Ic antiarrhythmic drugs). Moderate-intensity leisure-time sports activity can be resumed one week after ablation, and competitive activity after 1–3 months.

### 2.6. Premature Ventricular Contractions and Non-Sustained Ventricular Tachycardia

Premature ventricular complex (PVC) may be a sign of underlying cardiac pathology [34]. Specific features of PVCs such as origin (e.g., non-outflow tract or papillary muscle), high burden, complexity (e.g., couplets, triplets, non-sustained ventricular tachycardias), multifocal origin, and increased frequency during exercise should lead to the suspicion of structural heart disease. Athletes should also be thoroughly evaluated if PVCs are associated with baseline electrocardiogram abnormalities that point to an underlying cardiomyopathy. ECG in athletes reflects cardiac adaptations to exercise, and patterns potentially indicating cardiac diseases in sedentary subjects may be regarded as normal variants among athletes. Incomplete right bundle branch block and isolated increased QRS voltages meeting criteria for right or left ventricular hypertrophy criteria are especially common and should not lead to further evaluation. Another common normal variant, especially in athletes younger than 16 years of age or black/endurance athletes, is T wave inversion (TWI) in the anterior precordial leads (V1–V4) not associated with ST segment depression. TWI beyond V2 after age 16 warrants further assessment in Caucasian athletes. Similarly, TWI in lateral or inferior leads is uncommon in athletes and should raise suspicion of underlying cardiomyopathy. The presence of left bundle branch block always requires investigation [35]. In a single study, it was observed that 30% of athletes with more than 2000 PVCs per day had structural or familial heart disease [36]. In patients with frequent PVCs (>1000 PVCs/24 h) with normal ECG and echocardiogram, the presence of multifocal PVCs and a non-LBBB inferior axis pattern is strongly associated with the presence of CMR abnormalities (e.g., left ventricular wall motion abnormalities, presence of non-ischemic LGE, intramyocardial fat signal), which in turn are associated with an increased risk of fatal arrhythmias, especially a ring-like pattern of LGE. The presence of a family history of sudden cardiac death or cardiomyopathy is also an important “red flag” in the evaluation of athletes with PVCs. It has been suggested that the presence of ≥2 PVCs on a baseline ECG (or even ≥1 PVC in the case of high-endurance athletes) should prompt a more thorough evaluation, including a detailed family history. Among individuals with frequent PVCs and NSVT, a thorough investigation with 24 h ECG monitoring, 12-lead ECG, exercise stress test, and suitable imaging (e.g., CMR) is recommended [2]. In athletes with abnormalities found at an initial diagnostic work-up, the use of electroanatomical mapping (EAM) and endomyocardial biopsy (EMB), guided by EAM, is critical for obtaining a definite diagnosis [20]. All competitive and leisure-time sports activities are permitted, with periodic re-evaluation in individuals without familial or structural underlying disease.

### 2.7. Atrial Fibrillation and Atrial Flutter

Atrial fibrillation (AF) has a dual relationship with physical activity; moderate and regular sports practice is recommended to prevent AF; conversely, the prevalence of AF is increased by high-intensity endurance sports [2]. AF is not a contraindication to sports practice per se, but it point to underlying cardiomyopathies, with a potential impact on the assessment of sports eligibility. Furthermore, due to the heightened sympathetic tone during physical exertion, tests for both AF and atrial flutter (AFl) should be conducted for ventricles with high rates. For this reason, Class Ic antiarrhythmic drugs, which can slow atrial rate and thus facilitate 1:1 atrio-ventricular conduction, are not recommended in athletes [2]. The addition of β-blockers in order to control ventricular rate can have negative impact on physical performance, and β-blockers are even prohibited in some specific sports [2]. In this setting, catheter ablation can be the best choice for both AF (pulmonary vein isolation) and typical AFl (cavo-tricuspid isthmus ablation) [2]. As a final remark, among athletes requiring anticoagulant treatment because of the presence of risk factors for stroke, as per the CHA_2_DS_2_-VASc score (i.e., senior athletes with a high burden of concurrent cardiovascular risk factors), direct contact sports and sports associated to a high risk of trauma are prohibited [2].

### 2.8. Long QT Syndrome

Congenital long QT syndrome (LQTS) must be distinguished from the acquired forms. Congenital LQTS can be diagnosed in the presence of corrected QTc interval ≥ 480 ms according to Bazett’s formula on a 12-lead ECG, or a confirmed pathogenic LQTS mutation or a LQTS score >3 [37]. Patients with LQTS type 1 (LQTS1) are at the highest risk of cardiovascular events during exercise, especially swimming or diving [38]. Accordingly, asymptomatic athletes with LQT1 should not participate in sports that require immersion in water. Avoidance of medications that prolong the QT interval, electrolytic imbalances, and dehydration is critical. All athletes with LQTS with symptoms or prolonged QT should be on beta-blocker therapy. Patients who experience sudden syncope during beta-blocker therapy are candidates for ICD implantation or cardiac sympathetic denervation. Participation in high-intensity recreational and competitive sports, even when on beta-blockers, is not recommended in individuals with a QTc >500 ms or a genetically confirmed LQTS with a QTc >470 ms in men or >480 ms in women. Participation in competitive sports (with or without ICD) is not recommended in individuals with LQTS and prior cardiac arrest or arrhythmic syncope. In asymptomatic LQTS mutation carriers without a prolonged QT interval, i.e., <470 ms in men and <480 ms in women (‘genotype positive/phenotype negative’), shared decision-making is required, balancing the risk for arrhythmias with psychological well-being [2]. A negative exercise stress test is not sufficient to conclude that the patient is at low risk of SCD.

### 2.9. Brugada Syndrome

There is currently no evidence of increased arrhythmic risk during sports activity in athletes with Brugada syndrome. In asymptomatic individuals with Brugada syndrome, asymptomatic mutation carriers, and asymptomatic athletes with only an inducible ECG pattern, participation in sports activities that are associated with an increase in core temperature > 39 °C (e.g., endurance events under extremely hot and/or humid conditions) are not recommended. In athletes who have undergone ICD implantation for arrhythmic syncope or aborted sudden cardiac death and who have been asymptomatic for at least 3 months, return to sports is permitted with appropriate precautionary measures (i.e., padding the ICD generator, avoiding contact sports, and appropriate programming in order to avoid inappropriate shocks, see the section below).

### 2.10. Implantable Cardioverter Defibrillators and Pacemakers

The implantation of an ICD is not a contraindication to participation in sports activities per se, but rather it is the indication that the ICD (i.e., the underlying myocardial or electrical substrate) should be carefully reviewed at the time of sports eligibility assessment. Patients suffering from cardiomyopathies aggravated by moderate or high-intensity sports (e.g., in arrhythmogenic cardiomyopathy or lamin A/C mutations) should maintain restrictions on sports activity even if they have an ICD. In the athlete with an ICD, it is essential to optimize the device’s antitachycardia/shock therapy cut-offs in order to reduce inappropriate shocks.

Patients with pacemakers can participate in either recreational or competitive sports activity. However, it is recommended to properly program the pacemaker by carefully managing the upper rate limit and total atrial refractory period, in order to avoid sudden falls in heart rate during sports practice due to upper rate behavior [2]. In patients with pacemakers and a high percentage of ventricular pacing, the exercise stress test or ECG Holter during exercise can be useful to monitor pacemaker functioning [2]. Another aspect to consider is the protection of the device, both pacemakers and ICDs, and therefore avoiding sports that may lead to a high stress of the scapular girdle with a consequent risk of damage to the leads, as well as contact sports that can expose the shoulder region to blows, is recommended.

### 2.11. Congenital Heart Disease

Regular moderate intensity exercise is safe and effective in patients with congenital heart disease (CHD) [39]. Athletes with CHD should be considered as being at higher risk of sudden cardiac death during high intensity sports activity. Irrespective of type of defect, evaluation of these athletes should include a 12-lead electrocardiogram, 24-h ECG monitoring, including training period, and an exercise treadmill test. Depending on the burden of supraventricular and ventricular arrhythmias, QRS duration and fragmentation, and scarring due to previous cardiac surgery, CMR may be included in the evaluation. The presence of pulmonary arterial hypertension is a contraindication to competitive sports activity. Sport activity is also contraindicated in patients with aortopathy (e.g., aortic coarctation or bicuspid aortic valve) and aortic aneurysms > 5 cm. The presence of arrhythmias in patients with CHD may be the first indicator of haemodynamic deterioration, indicating the need for a complete re-evaluation. In athletes with CHD and symptomatic arrhythmias, an electrophysiological study is recommended.

## 3. Conclusions

In the practice of sports cardiology, it is essential to identify athletes at risk of SCD and to stratify their risk of major arrhythmias. Although the exercise stress test is the most widespread screening test in many countries, more advanced investigations may be required in specific settings (i.e., in athletes with suspected cardiomyopathies). According to the recent European guidelines on sport activity in patients with heart disease, a more liberal use of cardiac magnetic resonance imaging and electrophysiological study should be considered in many different conditions (Table 1), both in the risk stratification phase and in the phase of return to physical activity, whenever possible. On the other hand, the most relevant cause of SCD in athletes > 35 years old is ischemic heart disease, and therefore functional and anatomical tests for coronary artery disease should be largely employed in senior athletes. The assessment of athletes with suspected cardiovascular diseases should be done in referral centers with particular experience in the field, given the multiple challenges encountered.

## Author Contributions

Conceptualization, G.V., U.F., and P.C.; resources, M.C.; writing—original draft preparation, G.V., U.F., and P.C.; writing—review and editing, M.C., A.D.R., P.C., F.G., V.C., L.C., G.G., M.A.C., M.B., G.C., G.S., A.B., A.G., A.U., and M.F.; supervision, M.C., A.D.R., and P.C.; project administration, M.C. and A.D.R. All authors have read and agreed to the published version of the manuscript.

## Figures and Tables

**Table 1:** Multiparametric evaluation for risk stratification.

	EPS	CMR	Echocardiography	Exercise Stress Test	24 h ECG Monitoring	12 Lead ECG	Genetic Evaluation	Exercise Induce Symptoms	Previous Syncope
HCM		×	×	×	×	×		×	×
ACM		×	×	×	×	×	×	×	×
DCM			×	×	×	×	×	×	×
Myocarditis		×		×	×	×		×	×
WPW	×			×	×	×		×	×
PVC/NSVT		×	×	×	×	×			
AF/AFl			×	×	×	×			
LQTS					×	×	×	×	×
Brugada S	×				×	×			×
CHD	×	×			×	×			

## Data Availability

Data is contained within the article.
